# COVID-19 and Quality of Life: Twelve Reflections

**DOI:** 10.1007/s11482-020-09898-z

**Published:** 2021-01-06

**Authors:** Daniel T. L. Shek

**Affiliations:** Department of Applied Social Sciences, The Hong Kong Polytechnic University, Hong Kong, Hong Kong

**Keywords:** COVID-19, Reflections, Quality of life, Well-being

## Abstract

COVID-19 has severely affected the world since December 2020. Because of its sudden onset and highly contagious nature, the world has responded in a “crisis management” manner. With effective vaccines almost available, it is appropriate at this time to have some reflections about COVID-19 in relation to the quality of life issues. In this paper, we highlight twelve issues for reflection, which can help us better prepared for future pandemics. These include: digital divide, health inequality, gender inequality, economic disadvantage, family well-being, impact on holistic well-being, economic development versus saving lives, consumption versus environmental protection, individual rights versus collective rights, international collaboration versus conflict, prevention of negative well-being, and promotion of positive well-being.

COVID-19 has been with us for roughly 1 year. According to Johns Hopkins University, the number of infected cases is more than 65 million in early December 2020. With its sudden onset, highly contagious nature and widespread prolonged negative impact around the globe, governments, policy-makers, medical and allied professional as well as the general public have concentrated efforts to “put out the fire”. With the occurrence of different pandemic waves in different societies in the past year, there is not much time and space for reflections, particularly with reference to the different aspects of quality of life. In recent months, there is some good news that effective vaccines may be available soon, although the existence of effective vaccines does not mean that the problems can be resolved overnight. Hence, it is timely to have reflections about the impact of COVID-19 on quality of life so that we will be better prepared for the pandemic in the future. Examples from Hong Kong are highlighted for reflection.

## Reflection 1: Digital Divide

With city lockdown, people have relied more on online communication such as buying daily necessities via the Internet and work from home. Certainly, the efficiency of such activities depends very much on computer capability and smoothness of Internet connection, particularly for families in the rural area (Lai and Widmar 2020). For students, online teaching and learning has become the major form of education because of school lockdown. There are several advantages of online teaching and learning. First, teaching and learning mode is more flexible. Students can revisit the materials at times when they are convenient. Second, online pedagogies such as videos, virtual reality, games, and experiential learning activities are welcomed by young people who prefer to have active learning. Third, teaching and learning through online teaching can motivate and engage students. On the other hand, online teaching also creates several problems for students. First, if a student does not have a fast computer, online learning is a painful process. In Hong Kong, some students from poor families simply use the cell phones of the parents for learning, which creates health problems (such as eyesight and exercise problems) for them. Second, online learning depends on the availability of WIFI connection, which further depends on the financial resource of the family. Again, weak WIFI connection is a nightmare for online learning. Third, if students have more than one sibling in a family, there will be competition for computer use and WIFI data. If the parents also work from home, the competition would be even tougher. Finally, online learning reduces peer support and teacher guidance, which can typically be received during recess or lunchtime during normal school days. In a recent study, 43% of the teachers pointed out that there are communication and participation issues of online learning under COVID-19 (Stelitano et al. 2020).

Indeed, COVID-19 re-iterates the problem of digital divide or technological exclusion (Watts 2020), which adversely affects the academic well-being of students as well as family well-being. While some innovative initiatives have been carried out to reduce the digital divide under COVID-19 in Hong Kong, such as donating used computers for low-income families and financial support for subscribing to more stable WIFI service, the problem of online learning and unfavorable learning environment are issues to be further resolved.

## Reflection 2: Health Inequalities and Health Disparities

The pandemic has created two issues for the general public. The first one is prevention, such as keeping personal hygiene by wearing masks as well as using sanitizers. In the market, various products are available (ranging from surgical masks to N95 masks), and it depends on how much financial resource one has. This is an additional financial burden for people experiencing economic disadvantage. The second one is on treatment if one has been infected. There are variations in the medical treatment people can receive, such as using expensive drugs and staying in private wards. The quality of hospitalization varies significantly in different places, including Hong Kong.

There is research showing that the numbers of infected persons and deaths are not equal across different social groups and geographical areas. As pointed out by Norris and Gonzalez (2020), infected persons, hospitalized patients, and deceased cases are higher in “oppressed and disenfranchised” communities. Burström and Tao (2020) similarly remarked that COVID-19 had a more significant impact on people in the lower socio-economic class and minority groups. With reference to Africa, Okoi and Bwawa (2020) also showed that health inequalities adversely affected people’s responses to COVID-19.

## Reflection 3: Gender Inequality

Traditionally, women are regarded as caregivers in the family for those who are sick. As such, COVID-19 adds weight to the burden. Besides, students studying at home means more parental supervision, which is also commonly shouldered by women. In Hong Kong, some mothers from mainland China expressed the worry that they did not understand how to use laptops, such as downloading worksheets from online learning systems such as ZOOM. Empirically, phenomena on gender inequalities occur during COVID-19. Based on data collected from the United States, Germany, and Singapore, Reichelt et al. (2020) showed more work changes (including unemployment and drop-in work hours) in women than men. Power (2020) also pointed out that the invisible and unpaid care work of women increased under COVID-19. In the research area, Pinho-Gomes et al. (2020) indicated that there was gender inequality in research authorship in COVID-19 studies where women researchers were under-represented.

There are several areas for us to reflect on gender inequalities. First, it is essential to consider how to involve fathers more in family tasks, particularly caregiving tasks. We need to change the prevailing beliefs about the role of fathers in the socialization process. Second, changing the culture on the involvement of men in the family is important. Essentially, we have to reflect on empowering women so that the family responsibilities will not fall solely on them. Third, more awareness of the importance of gender equality should be promoted. Finally, we should develop appropriate services to support women under the pandemic.

## Reflection 4: Intensification of Poverty

While COVID-19 creates growing opportunities for some industries such as Internet-based purchase, electronic games and health care materials, it has adversely affected the global economy, especially transportation, tourism, catering, where many semi-skills and unskilled workers are involved. Although the Government has launched two rounds of job support schemes in Hong Kong, many workers have been forced to take no-pay leave. For some airline industries in Hong Kong such as Cathy Pacific and Dragon Air, massive layoffs happened which lead to unemployment problem. Psychologically, unemployment creates increased stress and mental health problems for unemployed persons. Such problems will spill over to marital quality, which would further adversely affect family processes such as parenting and family functioning processes. For communities with a large number of low-income families, community cohesion is typically not high, and there are many social problems such as crime and health issues. Hence, minimizing poverty arising from COVID-19 is an important policy priority. While many countries have job support schemes, the difficulty is that the duration cannot be too long due to constraints in economic resources. Relevant services supporting unemployed people and their families should also be considered.

Sumner et al. (2020) estimated the impact of COVID-19 on poverty indexed by per capita household income and consumption and concluded that COVID-19 could increase global poverty and constitute obstacles to attaining the goal of eliminating poverty by 2030. Assuming consumption contraction of 20%, they also estimated an increase of 420–580 million poor people compared to the 2018 figures. Buheji et al. (2020) also remarked that COVID-19 is a “new source” of poverty that has a negative economic impact on developed and under-developed economies. As such, Pak et al. (2020) argued for the “epidemic preparedness” concerning the economic consequences of pandemic.

## Reflection 5: Family Well-Being

COVID-19 has tremendous consequences on family well-being. In the first place, city lockdown means family members will have more time to stay at home. While this may promote family cohesion and provide more opportunities for interaction amongst the family members, staying at the family may pose at least two challenges. First, if family members have different views, prolonged stay in the family would create more conflicts. For example, staying at home during COVID-19 in Hong Kong creates additional conflicts for family members because parents and their children may hold different political views about the Social Event taking place in 2019–2020 (Shek 2020b). Besides, if the space in the family is small, staying at home means less personal space for each family member, and they also compete for family resources such as WIFI data and space. In the case of Hong Kong, as household space is typically small and children do not have single rooms, family conflicts are likely to happen.

The second challenge is related to parenting. If parents and their children stay at home, parental supervision burdens increase, such as supervising their children to use the computer and asking them to follow a healthy daily routine. Besides, because of school lockdown, students study via online learning. This learning mode also demands parental involvement. This would be a challenge for parents who are not familiar with computer usage and online learning procedures. As mentioned, many mothers of disadvantaged groups have highlighted this problem in Hong Kong. COVID-19 also poses a threat to the work of the parents resulting in economic strain for the parents and the family. These problems would be more pronounced if family resilience in the family is not strong.

Undeniably, families are shouldering additional responsibilities under COVID-19. Fisher et al. (2020) pointed out that “the COVID-19 pandemic has forced families to try to maintain work-family balance with few supports. With schools and daycare facilities closed, parents are solely responsible for childcare and perhaps even homeschooling” (p.249). Similarly, Gromada et al. (2020) pointed out that COVID-19 created additional childcare tasks for families and suggested that additional measures such as paid leave and high-quality non-family childcare services should be introduced. Janssen et al. (2020) found that while most parents were able to cope well with family demands, there was an increase in the negative effect of parents with variation in the related impacts in different families.

## Reflection 6: Holistic Quality of Life

COVID-19 is a physical disease. As such, public attention has been placed more on the numbers of infected cases and death tolls as well as physical health complications arising from the disease. While it is crucial to focus on the physical well-being consequences of COVID-19, looking at other aspects of well-being under COVID-19 is equally important. In the medical and allied professionals, it is commonly argued that there are four domains of health, including physical, psychological, social, and spiritual domains. Besides the physical consequences of COVID-19, we need to understand the psychological consequences of the pandemic, such as depression and post-traumatic stress disorders. Most importantly, it is vital to understand how different psychological resources such as adversity quotient (AQ), emotional quotient (EQ), and coping resources may help individuals cope with stress arising from COVID-19. Unfortunately, the focus on the non-physical aspect of well-being is not strong in many places, including Hong Kong.

Regarding social health, there are several areas of concern. First, as mentioned above, there are positive and negative consequences of COVID-19 on families. Hence, how to maintain family health and promote family resilience is an important question to be addressed. One argument we should consider is that the promotion of family social capital is an attractive option for the family with a reduction in the financial capital of the family under COVID-19. Second, with city lockdown, social interaction drops. As social support is a protective factor of adversity, reduction in social interaction is a threat. Undoubtedly, with Internet technology, it is possible to maintain social contact with others. However, Internet technology may be a challenge for old people who are commonly unfamiliar with the latest Internet technology. In Hong Kong, many aged people are still using 3G cell phones. The same social well-being challenge exists for young people. During school lockdown, young people can only count on online interaction with others. While some may over-use such mechanisms, others may feel lonely if they have difficulty in WIFI connection. Finally, relative to studies on physical well-being, comparatively fewer studies have been conducted to examine spiritual well-being under COVID-19. Focus on spiritual well-being is essential for two reasons. First, finding meaning in suffering under COVID-19 is vital. Second, spiritual resources such as seeking help from God and having positive beliefs about resilience (such as positive cultural beliefs about adversity) are important for positive coping.

## Reflection 7: Economic Growth Versus Saving Lives

COVID-19 has led to the slowdown of the global economy. Because of city lockdown, many industries have been adversely affected, such as the catering, hotel and tourism, and airline industries. As economic growth is a key performance indicator of a government and many small and medium-sized businesses will die with prolonged shutting of the economic engine, there is a cry for restarting the economic engine by governments. For example, Donald Trump indicated his wish to restart the economy after Easter 2020. In some European countries in summer 2020, officials devised plans to save the tourism and hotel industries when the number of infected cases decreased. On the other hand, there are views cautioning restarting the economy prematurely, which will increase the death toll and the number of infected cases. In Hong Kong, there is a continuous debate on this issue. While it is commonly agreed it is vital to save lives, the economic price of saving lives may not be widely endorsed.

Hence, the fundamental reflection point here is whether to seek economic growth (which will increase human encounter) or to save lives by reducing economic activities (which will decrease human encounter). Obviously, there are different views of different stakeholders. While businesspeople and workers with income tied with the performance of the economy would urge for a fast restart of the economy, health professionals and those with health risk would favor a more cautious approach in restarting the economic engine. Hence, more reflections on this issue and collection of public opinion are important.

## Reflection 8: Consumption Versus Environmental Protection

One unintended consequence of COVID-19 is that it provides us a golden opportunity to re-think the issue of consumption versus environmental protection. With city lockdown and substantial reduction of air travel, COVID-19 has brought some good news for environmental protection. Le Quéré et al. (2020) showed that because of confinement in various places, the peak reduction in carbon dioxide emissions was 26%. Liu et al. (2020) also reported a drop of 8.8% of carbon emissions in the first half of 2020 compared to the same period in 2019. Shakil et al. (2020) summarized the impact of COVID-19 on the environment: while lowered air and sound pollution, temperature, and humidity were related to COVID-19, medical and domestic wastes increased. Klenert et al. (2020) pointed out that “learning from policy challenges during the COVID-19 crisis could enhance efforts to reduce GHG emissions and prepare humanity for future crises” (p. 751). Arora et al. (2020) asserted that with the shutdown of diverse types of activities, “nature takes the advantages and showed improvement in the quality of air, cleaner rivers, less noise pollution, undisturbed and calm wildlife” (p.1). They even concluded that “although coronavirus vaccine is not available, coronavirus itself is earth’s vaccine and us humans are the virus” (p. 1).

With environmental repair under COVID-19, there are several questions one should reflect upon. The first question is whether it is necessary to have so much air travel related to business and pleasure. With COVID-19, many business meetings are going online. Similarly, people begin to realize that they can live happily (or at least healthily) without taking trips via air travel. The second question is whether there is a need to change our lifestyle, such as reducing unnecessary consumption. We must ask ourselves two questions: a) do we need to consume so much? b) can we enjoy well-being without excessive material consumption? Under Capitalism, consumption is the driver for economic growth, creating “wealth” for people. Also, consumption is encouraged under Capitalism because it helps to maintain the circular flow of income. However, it is time to reflect upon the price we have to pay for endless economic activities and explore whether there are more environmentally friendly options.

## Reflection 9: Individual Rights Versus Collective Rights

COVID-19 has generated an interesting but prominent issue regarding the importance of individual rights versus collective rights (Mykhalovskiy et al. 2020). For example, Toebes (2020) pointed out there was tension between individual rights and public health and whether government measures would compromise human rights and personal privacy. Regarding the proposal to wear masks, scientific findings show the preventive role of wearing facial masks (Li et al. 2020), and wearing masks can be regarded as a sign of altruism which can promote social solidarity (Cheng et al. 2020). However, some people do not follow the policy of wearing facial masks because they believe this is an infringement of the individual right. One interesting observation is that while many countries and places have a policy of requiring people to wear masks in public places, resistance to such a policy is much stronger in Western societies than in Asian societies.

## Reflection 10: International Collaboration Versus International Competition

Faced with the global pandemic, it is reasonable to encourage international collaboration. However, there is politics in international collaboration (McKenzie 2020), as shown by the occurrence of several unfortunate events. First, blaming the victim formed the international discourse in the first few months. Although people in Wuhan suffered from the pandemic, they were blamed for spreading the virus. Political figures in several countries claimed that COVID-19 is a “Chinese virus” and they even demanded compensation. With reference to available scientific findings, scientists have clearly pointed out that there is no concrete conclusion on the origin of COVID-19, and traces of coronavirus were found in a sewage sample in Spain in March 2019. Second, although the World Health Organization has tried its absolute best to cope with the pandemic, it was criticized heavily for the “slow” response and not making impartial responses. Third, different countries and societies tend to develop their own measures to cope with the pandemic without much international concerted efforts. Susskind and Brown (2020) argued that infectious diseases control such as COVID-19 should be treated as “global public goods”, and international cooperation is key to the successful implementation of the related tasks. Fry et al. (2020) pointed out that smaller teams, narrowed membership, elitism and fewer societies were involved in COVID-19 research compared to pre-COVID research in coronavirus. Considering the high failure rate of development COVID-19 research, Zhou (2020) argued for the stepping up of effort for international collaboration involving different stakeholders should be promoted. Finally, Basrur and Kliem (2020) argued that while different approaches offer insights on international cooperation, the realistic perspective highlighting national interests over collective effort is a useful tool to understand international collaboration.

## Reflection 11: Prevention of Negative Well-Being

In view of the negative impacts of COVID-19 on different well-being domains, the question of how to prevent COVID-19 and its negative well-being consequences is in order. From the public health perspective, there are at least two strategies one should adopt (Lu et al. 2020; Shek 2020a). The first strategy is minimization of risk factors associated with the negative impacts of COVID-19, such as helping disadvantaged areas with less-developed medical services and social groups with inadequate health education. The second strategy is to strengthen factors that can protect people under COVID-19 (i.e., protective factors). With particular reference to young people, findings support the importance of developmental assets in promoting well-being in young people, such as resilience, emotional competence, spirituality, and self-efficacy (Ma et al. 2019; Shek and Zhu 2020; Zhu and Shek 2020). It is suggested that validated positive youth development programs, particularly those nurturing life skills in young people (Shek et al. 2020), would help young people cope with adversity. Apparently, having prevention initiatives before the pandemic would better prepare people for the trauma associated with the pandemic, and such initiatives can save lives.

## Reflection 12: Maintaining Positive Quality of Life under COVID-19

The final reflection is concerned with the recipe for maintaining positive quality of life under COVID-19. Although there are different answers based on different theories, several factors contribute to positive quality of life. First, an accurate understanding of COVID-19 and how to prevent it (such as maintenance of personal hygiene) can reduce the perceived stress surrounding COVID-19. Second, maintaining hope is always a key factor under adversity. One way is to look at the bright side of the pandemic (there must be some). Third, maintaining social contact provides support and assistance under the pandemic, particularly social capital generated within the family context. Fourth, finding the meaning of suffering under COVID-19 is helpful because purpose shapes attitude to the pandemic and coping repertoires. Fifth, instead of over-criticizing the governments for their inability to stop the pandemic, showing appreciation of the work done by government officials, medical and allied professionals, teachers teaching online, volunteers, and the general public who adhere to the health restriction measures can help to generate a positive community culture. Learning how to be grateful to those who have served under COVID-19 is also important. Sixth, empowerment for oneself, peers, family, community, and society levels is helpful. Finally, having a resilient mindset (i.e., regarding pandemic as a chance for growth and development), embracing the challenges and treating them as opportunities to grow (Yamaguchi et al. 2020) can help to promote positive well-being under COVID-19.

The human race has encountered several pandemics in the past centuries. Successive plagues related to the Black Death in Medieval Europe (such as the Black Death Bubonic Plague from 1347 to 1351) killed many people in Europe and the near East. Almost a century ago, the Spanish flu also killed millions of people throughout the globe. In these two pandemics, human beings were very helpless and powerless. With technological advances, we are in a better position to understand the genetic makeup and properties of COVID-19 coronavirus. When effective vaccines are almost ready, it is timely to reflect on the issue of quality of life related to the pandemic.
